# Lost to follow-up while undergoing intravitreal injection therapy: barriers to adherence in a real-world setting

**DOI:** 10.1186/s40942-026-00808-3

**Published:** 2026-02-11

**Authors:** Annika Licht, Yiannis Mavris, Catharina Latz, Alexander K. Schuster, Katharina A. Ponto, Alireza Mirshahi

**Affiliations:** 1 Dardenne Eye Hospital, Bonn, Germany; 2 Department of Ophthalmology, Johannes Gutenberg-University, Mainz, Germany

**Keywords:** Intravitreal injections, anti‑VEGF, Adherence, Persistence, Lost to follow‑up, LTFU, Neovascular age‑related macular degeneration, Diabetic macular edema, Retinal vein occlusion, Real‑world evidence

## Abstract

**Background:**

Intravitreal anti‑VEGF therapy is effective, yet real‑world outcomes are limited by non persistence. We investigated patient‑reported reasons for discontinuation under pro-re-nata therapy protocol.

**Methods:**

This ambispective real-world study at a German eye hospital evaluated patients receiving intravitreal therapy for diabetic macular edema (DME), retinal vein occlusion (RVO), or neovascular age related macular degeneration (nAMD) using the pro re nata (PRN) protocol. Patient records were reviewed, and those meeting lost to follow up (LTFU) criteria (no injections or visits within 3 months) were contacted via written letter and phone. When patients were reached surveys to assess reasons for LTFU, perceived treatment burden, mobility, and comorbidities were completed.

**Results:**

Of 657 cases, 347 (52.8%) met the LTFU- criteria, 25 of which were medically indicated. Despite multiple attempts, contact and data collection was successful for only 39 patients (response rate: 12%), major part was unwilling or unable to participate. The most common reasons for discontinuation were subjective lack of treatment need (56%), perception of being “fully treated” (33%), fear of injections (26%), travel difficulties (28%), and time burden (15%). Despite these barriers, 52% of patients reported that the treatment was “not burdensome at all,” while 3 patients (8%) continued therapy elsewhere.

**Conclusions:**

In this exploratory, single-centre cohort, a significant portion of patients had effectively disengaged from IVI care and were unreceptive to delayed outreach. Outcome-related perceptions and organizational hurdles were key, actionable barriers. Future strategies to improve adherence should focus on proactive follow-ups shortly after therapy discontinuation.

**Supplementary Information:**

The online version contains supplementary material available at 10.1186/s40942-026-00808-3.

## Introduction

Intravitreal injection (IVI) of vascular endothelial growth factor (VEGF) inhibitors is widely used to manage vision-threatening eye diseases, including diabetic macular edema, retinal vein occlusion, and neovascular age-related macular degeneration [1–4] . While clinical trials demonstrate superior outcomes in efficacy and application, real-world studies often reveal less favorable results [5, 6].

This discrepancy may be attributed to insufficient adherence, as the effectiveness of IVI therapy is closely tied to these factors [7, 8]. Differences between trial injection frequencies (up to 12/year) and routine care (often 4–5/year) have been described [9], underscoring the relevance of adherence and expectation management.

Frequent 4-weekly treatment and monitoring appointments impose a significant burden on patients, increasing the risk of non-adherence [10, 11]. To address these challenges, flexible treatment protocols such as Pro Re Nata (PRN) and Treat-and-Extend (T&E) have been developed.

Nevertheless, adherence and persistence remain suboptimal even with these strategies [12]. LTFU, including missed injection and follow-up appointments, is a key risk factor for poor visual outcomes [6, 11, 13].

This study aims to quantify LTFU under PRN injection scheme and investigate patient-driven factors for therapy discontinuation under real-world conditions. Understanding these factors, including fear of injections, logistical challenges, and the impact of comorbidities, is essential to improve care delivery and patient outcomes in ophthalmology.

## Methods

### Study design

Site procedures. All IVIs were performed under topical anesthesia after povidone-iodine antisepsis using a 30-gauge needle at a 3.5 mm measured distance from the limbus. Post-injection prophylaxis was not routinely prescribed. Typical visit duration at our site was approximately 60 minutes for injection visits and 30 minutes for follow-ups. The PRN protocol used functional (best correlated visual acuity (BCVA)) and anatomical (optical coherence tomography (OCT)-based Central retinal thickness (CRT)) criteria for re-treatment. No automated appointment reminders were implemented during the study period.

This study employed an ambispective cohort design to identify patients who began intravitreal injection (IVI) therapy for diabetic macular edema (DME), neovascular age-related macular degeneration (nAMD), or retinal vein occlusion (RVO) with macular edema at our eye clinic between January 2018 and December 2019 and subsequently discontinued therapy. All patients were treated according to the pro re nata (PRN) protocol. Therapy discontinuation was defined as the absence of injections or follow-up appointments for a period exceeding three months, with no resumption thereafter until May 2021.

### Data collection

**Fig. 1:**
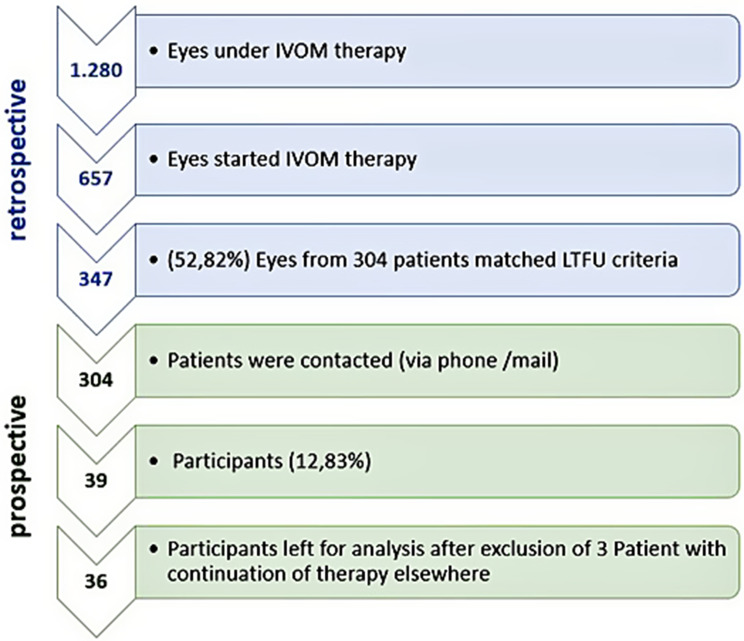
Study design and participants

During the prospective phase, patients who met the criteria for treatment discontinuation were contacted by phone (up to three attempts) or by mail and invited to complete a structured questionnaire. Data from treatments administered between January 1, 2018, and December 31, 2019, were analyzed. The data were first accessed on August 23, 2021, for research purposes. The recruitment period began on September 1, 2021, and concluded on May 12, 2022. Study design and participation can be seen in Fig. 1. In case of non-participation, the reason for non-participation was documented as shown in Table 1.

**Table 1:** Reasons for non-participation in the questionnaire study

Reasons for non-participation		
Not available/no response	118	45%
Deceased	23	9%
Relocation/continuation of treatment elsewhere	29	11%
no response/interest/language problem	80	30%
Other illness	14	5%
Never received injections	1	0%
Total	265	100%

The ambispective study design was chosen to adequately address the research objectives under consideration of real-world clinical conditions. The retrospective component was necessary to systematically identify and characterize patients treated under the PRN regimen and to determine therapy adherence and discontinuation status based on routinely collected clinical data. Only after this structured identification process could eligible patients be defined and grouped according to therapy status.

The subsequent prospective patient contact and questionnaire-based assessment therefore necessarily occurred with a temporal delay.

The questionnaire assessed:

1. Therapy Status: Whether the patient had continued IVI therapy at another location.
2. Reasons for Discontinuation: Patients selected reasons from a predefined list and rated their importance on a Likert scale from 1 (decisive) to 4 (less relevant).
3. Treatment Burden: Questions addressed pain or discomfort during treatment, satisfaction with ongoing therapy, and willingness to recommend IVI therapy. Duration and overall satisfaction with treatment were also evaluated.
4. Appointment Organization: Patients were asked about the burden of keeping appointments, missed appointments, and reasons for missing them (e.g., lack of transportation, other illnesses, or forgetting).
5. Mobility and Accessibility: Patients provided information on transportation modes, the role of support persons during appointments, travel time to the clinic, and whether a health-insurance-provided taxi voucher would influence therapy continuation.
6. Comorbidities: Patients were queried about the presence of comorbidities and their associated scheduling burden.

## Results

### Survey participation

657 eyes began intravitreal injection (IVI) therapy for nAMD, DME, or RVO with macular edema at our facility between January 2018 and December 2019. 347 eyes of 304 patients met the criteria for therapy discontinuation. Of these 304 patients, 39 patients participated in the survey (response rate: 12%). The rate of patients who temporarily interrupted therapy and resumed it after May 2021 was not assessed.

Reasons for non-participation among the remaining 265 patients in the survey were as follows: 22 patients reported continuing therapy at another facility, 14 prioritized another medical condition, 90 were unreachable, 28 did not return contact attempts, and 51 were uninterested in participating. Twenty-three patients were documented as deceased, and six cited relocation (three moved homes, and three entered care facilities) as the reason for non-participation.

### Patient demographics

Among the 39 survey participants, nine received IVI therapy in both eyes, while 30 were treated for one eye. Participants ranged in age from 47 to 93 years (mean: 74 years), and 56% (*n* = 22) were male. After excluding three participants who continued therapy elsewhere, 36 patients were included in further analysis. Among them, 39% (*n* = 14) had DME, 50% (*n* = 18) had nAMD, and 11% (*n* = 4) had RVO with macular edema.

### Reasons for therapy discontinuation

Discontinuation of therapy was reported as follows: 61% (*n* = 22) of participants stated “no further need for therapy,” and 36% (*n* = 13) reported being “fully treated.” Both statements were intended to reflect the subjective perception that continuation of therapy would provide no personal benefit.

A further 28% (*n* = 10) of responses indicated fear of intravitreal injection (IVOM) as a reason for premature discontinuation of therapy.

Seventeen percent (*n* = 6) reported “other reasons” without further specification. Fourteen percent (*n* = 5) cited “long travel distance,” 11% (*n* = 4) “high time burden,” 8% (*n* = 3) “lack of transportation,” and 6% (*n* = 2) “pain.” Each of the following reasons was reported by 3% (*n* = 1): “burdensome follow-up care,” “lack of support,” and “not wanting to be a burden.”

Seventeen of 36 participants weighted their responses. The most frequently cited reason for discontinuation was perceived treatment outcome (weighted 47%), with patients reporting no subjective need for continued therapy or considering themselves fully treated. Organizational factors, including time burden and travel distance, represented the second most common category (28%). Fear of injections was also frequently reported (26%). Detailed responses are presented in Table 2.

**Table 2:** Reasons for therapy discontinuation (weighted multi response)

Answer	Frequency (weighted)	Frequency Group
Fear of injection	13%	**Treatment issues**
Pain	3%	16%
Time-consuming aftercare	1%	
Lack of transport	3%	
Lack of support from relatives/friends	3%	
Not wanting to be a burden on relatives	2%	**Organizational issues** 28%
Time required	10%	
*Financial burden*	0%	
distance to the clinic/practice too far	9%	
No further need for therapy	29%	**Outcome-related**
Treatment completed	18%	47%
Other reason	9%	**Other** 9%

Further analysis of visual acuity improvement under IVOM therapy was conducted among participants who reported a subjective lack of treatment benefit. The mean change in visual acuity among patients who did not expect subjective benefit from further therapy was below 0.1 in all groups. The highest gain was observed in patients with AMD (0.07), whereas changes in patients with DME and RVO were 0.04 and 0.05, respectively. No improvement was observed in the group of patients with diabetes, which constituted the largest subgroup (13/24, 54% of respondents). Two participants who considered themselves “fully treated” (both with DME) attended only a single IVOM visit; no follow-up visual acuity data were available.

#### Treatment burden

Regarding treatment burden, 53% of participants (*n* = 19) reported feeling “not burdened at all,” while 33% (*n* = 12) felt “somewhat burdened.” Most participants expressed motivation to continue therapy if medically necessary: 58% (*n* = 21) were fully motivated and 19% (*n* = 7) moderately motivated. Additionally, 72% (*n* = 26) of participants responded the “fully recommended” the therapy, 17% (*n* = 6) responded “somewhat yes,” and only one participant responded “rather no.”

Treatment appointment duration was reported as less than two hours by 50% of participants and between two to four hours by 39%. Overall, 80% (*n* = 29) were satisfied with the time required for treatment.

### Appointment organization

Injection appointments were considered “not burdensome at all” by 83% (*n* = 30) of participants, while attending these appointments was rated “somewhat burdensome” by 19% (*n* = 7) and “very burdensome” by 14% (*n* = 5). Follow-up visits were considered “not burdensome” by 75% (*n* = 27) and “very burdensome” by 8% (*n* = 3). Most participants (78%, *n* = 28) reported not receiving appointment reminders from the clinic.

### Missed appointments

Overall, 8% (*n* = 3) of participants reported missing a single injection appointment, and 3% (*n* = 1) reported missing multiple appointments. In total, four patients indicated that they had missed at least one appointment, one of whom missed appointments repeatedly. Reasons included forgetting the appointment (“simply forgot,” AMD patient), other illness (AMD patient), time-related issues (DME patient), and other unspecified reasons (two DME patients).

### Mobility and transportation

The most common mode of transportation was by private car (68%, *n* = 27), followed by public transportation (28%, *n* = 11). Four participants used multiple modes of transport. Support from family members or companions was required by 81% (*n* = 29) for injection appointments and by 39% (*n* = 14) for follow-ups. Spouses (*n* = 12) and children (*n* = 8) were the most frequent companions, followed by friends (*n* = 6) and others (*n* = 2).

The majority of participants did not require assistance for follow-up visits (22/36), particularly patients with DME (13/18). However, 14 participants required support for follow-up appointments, predominantly patients with AMD (7/14).

Nearly all participants required assistance to attend injection appointments. This included almost all patients with AMD and RVO (13/14 AMD patients and 3/4 RVO patients) and the majority of patients with DME (13/18), while five DME patients reported not requiring assistance.

Regarding reimbursement of travel costs by health insurance, most participants (15/35, 43%) stated that this would “not influence at all” their decision to discontinue therapy. Twenty-three percent were undecided, and 17% reported that it would “rather not” influence their decision. Only 14% (5/35 participants) indicated that reimbursement would “fully” influence their decision to continue therapy.

### Travel time

Travel time to the injection center was reported as 30–60 minutes by 56% (*n* = 20), over 60 minutes by 22% (*n* = 8), and under 30 minutes by 22% (*n* = 8). For follow-up appointments, 67% (*n* = 24) reported travel times of less than 30 minutes, 25% (*n* = 9) reported 30–60 minutes, and 8% (*n* = 3) reported over 60 minutes.

### Comorbidities

Ninety-two percent (*n* = 33) of participants reported having comorbidities requiring regular medical attention. The most common comorbidities were diabetes mellitus (*n* = 15), cardiovascular disease (*n* = 11), hypertension (*n* = 14), joint diseases (*n* = 10), thyroid disorders (*n* = 4), and other conditions (*n* = 3).

Only a small number of participants had no comorbidities (3/36), predominantly patients with AMD and one patient with RVO. An equal number of participants reported having one or two comorbidities (*n* = 13 each). Among those with one comorbidity, five were patients with AMD and eight with DME. Among those with two comorbidities, five were patients with AMD, six with DME, and two with RVO. Multimorbidity, defined as three or more comorbid conditions, was present in seven participants (19%), including two patients with AMD, four with DME, and one with RVO.

Regarding the frequency of doctor visits, 58% (*n* = 21) visited less than once every four weeks, 19% (*n* = 7) visited every four weeks, 3% (*n* = 1) visited every two weeks, and 14% (*n* = 5) did not have regular doctor´s appointments.

## Discussion

### Principal findings

Discontinuation of intravitreal injection (IVI) therapy was multifactorial [6]. The strongest driver in our data was the patient’s subjectively perceived treatment outcome—especially the perception that treatment was “completed” or that there was “No further need for therapy”. After ≥1 year, many patients could not/would not specify reasons. The high LTFU and low response rates may be attributable to delayed recall, as telehealth-based recall has been identified as a key strategy to reduce LTFU in patients with AMD [14]. To the date of this study, appointment reminders were not included in our treatment procedure.

### Perceived benefit as the dominant patient-reported reason

The most frequently selected reasons were “no further treatment necessity” (61% [22/36]) and “treatment completed/no further need for therapy” (subgroup; multiple answers possible). These responses reflect subjective benefit/necessity judgements and do not, on their own, explain why discontinuation occurred. In the ““treatment completed/no further need for therapy”” subgroup, mean visual acuity change was small (overall ~0.01; AMD ~0.10, DME ~0.00, RVV ~0.04); follow-up acuity was partly missing, two patients only visited one injection appointment.

Subjective lack of benefit was cited by 67% overall (AMD 50% [*n* = 7], DME 72% [*n* = 13], RVE 100% [*n* = 4]); in this subset, no patient improved by ≥0.1 decimals (maximum AMD 0.07). Similar reasons (no improvement/low perceived benefit) are known drivers of LTFU/nonadherence in anti-VEGF care: With mean baseline visual acuity 0.4, this pattern is consistent with prior reports on discontinuation in association with low perceived benefit [15, 10, 16, 17]; found “no improvement in vision” as second most reason for LTFU.

Our data support the view that patients’ subjective perspective is central for adherence [9]. Communication should emphasize stabilization and prevention of deterioration when improvement is not perceived. Good visual acuity is correlated with perceptions of effectiveness [18].

### Contextual factors and limitations

Logistical burden was common: most respondents needed support, including accompaniment during the visit (25 participants), yet none reported missing an appointment due to lacking support. Travel-cost reimbursement was mostly rated as non-influential (43% “not at all”, 17% “rather not”, 23% undecided), but crucial for some of the LTFU-Patients (14% “fully” influential). Our hospital provides transportation support, but this might be extended to include more patients.

Comorbidity was frequent, but was rarely linked to missed visits (one AMD respondent). Given small subgroups, our data could not confirm comorbidity-related assumptions with LTFU reported for DME [19, 20] or more common frailty AMD [21].

Procedure-related concerns were less prominent, but persistent: fear/anxiety was named as reason for LTFU by 28% (10/36), pain 6% (2/36).

### Protocol considerations

This cohort was treated under a Pro Re Nata (PRN) protocol [22]. Treat-and-Extend regimens and newer agents have been reported to improve visual outcomes over time and may reduce injections per year [23, 24] our results provide a baseline for comparing adherence and satisfaction across protocols and medication eras.

## Limitations

Although this study is limited by its small sample size and the subjective nature of participant responses, it provides valuable insights into patients’ perceptions, even up to two years after they were lost to follow-up. These insights emphasize the importance of patients’ understanding of their condition and treatment in improving therapy adherence. We recommend further research that compares these patients’ experiences with those of patients receiving treatment under the “treat and extend” protocol.

## Conclusion

In this cohort from a German eye hospital, most LTFU patients could not be contacted or declined participation. Among respondents who provided feedback, the most frequently reported reason for discontinuation was the subjective impression that treatment would be without additional use or has been completed, accompanied by additional concerns such as fear of injections and organizational burden. These findings indicate the importance of clear communication regarding long-term treatment goals and of structured early follow-up after treatment interruptions to support adherence.

### Practical implications

- Subjective treatment outcomes are pivotal in patients’ decisions to discontinue therapy. Clear communication about the benefits of ongoing treatment, including the value of vision stabilization, should be emphasized during patient interactions.
- Organizational challenges, such as travel and appointment burdens, contribute to patient dissatisfaction. Individualized solutions, including combined scheduling and logistical support, could enhance adherence and improve long-term outcomes.
- All authors approved the final version of the manuscript and agree to be accountable for all aspects of the work.

## Electronic supplementary material

Below is the link to the electronic supplementary material.

Supplementary material 1

## Data Availability

The datasets analyzed during the current study are not publicly available due to patient confidentiality regulations but de-identified data are available from the corresponding author on a reasonable request.
