# Supplementary material for: Lost to follow-up while undergoing intravitreal injection therapy: barriers to adherence in a real-world setting
Source: Int J Retina Vitreous. 2026 Feb 11;12:39. doi: 10.1186/s40942-026-00808-3 (PMC12927251; doi:10.1186/s40942-026-00808-3)
Supplement: Supplementary file 1 — Supplementary material 1 [file 40942_2026_808_MOESM1_ESM.docx]

**Questionnaire**

**Patient questionnaire to determine reasons for discontinuation of therapy and factors influencing adherence**

Date: ___________

Patientencode:________________________

1. **Therapy status**
2. You have started intravitreal injection therapy with us. Have you continued the therapy elsewhere? ☐No ☐Yes ☐Don't know ☐No answer

| 1. What was the decisive reason(s) for you not to continue therapy? You did not continue therapy because… | No | Yes |
| --- | --- | --- |
| - 1. … you were afraid of the injection? |  |  |
| - 1. … you had pain during / after injection? |  |  |
| - 1. … the aftercare was too time-consuming? |  |  |
| - 1. … you had no transport options? |  |  |
| - 1. … you had no support from relatives or friends? |  |  |
| - 1. … you did not want to be a burden on your relatives or friends? |  |  |
| - 1. … the time required was too long? |  |  |
| - 1. … the financial burden was too long? |  |  |
| - 1. … the distance to the practice/clinic was too far? |  |  |
| - 1. … there was no longer a need for a therapy? |  |  |
| - 1. … the treatment has been completed. |  |  |
| - 1. Other reason |  |  |
| - 1. Don't know |  |  |
| - 1. No answer |  |  |

If you had to prioritize the reasons, in which order would you name the reasons just listed (1=decisive; 4=less decisive)?

(1)

(2)

(3)

(4)

1. **Stress caused by treatment**
2. How much discomfort or pain did you feel during treatment?

|  | Not burdened at all |  | Somewhat burdened |  | significantely stressed |  | Fully and completely burdened |  | Don't know |  | No answer |
| --- | --- | --- | --- | --- | --- | --- | --- | --- | --- | --- | --- |

1. If further treatment is required, how satisfied would you be with continuing or repeating the treatment?

|  | Fully and completely satisfied |  | Fairly satisfied |  | Somewhat satisfied |  | Not at all satisfied |  | Don't know |  | No answer |
| --- | --- | --- | --- | --- | --- | --- | --- | --- | --- | --- | --- |

1. How satisfied are you with the time you spent in the clinic on the day of treatment?

|  | Fully and completely satisfied |  | Fairly satisfied |  | Somewhat satisfied |  | Not at all satisfied |  | Don't know |  | No answer |
| --- | --- | --- | --- | --- | --- | --- | --- | --- | --- | --- | --- |

1. On average, how much time did you spend in the clinic on the day of treatment?

less than 2 hours

2-4 hours

more than 4 hours

Don’t know

No answer

1. How satisfied are you with the overall duration of the treatment?

|  | Fully and completely satisfied |  | Fairly satisfied |  | Somewhat satisfied |  | Not at all satisfied |  | Don't know |  | No answer |
| --- | --- | --- | --- | --- | --- | --- | --- | --- | --- | --- | --- |

1. Would you recommend this type of treatment to another person?

|  | Fully and completely |  | Rather yes |  | Rather no |  | Not at all |  | Don't know |  | No answer |
| --- | --- | --- | --- | --- | --- | --- | --- | --- | --- | --- | --- |

1. **Organisation of appointments and treatment**
2. How time-consuming was it for you to attend the follow-up visits to the ophthalmologist?

|  | Not time-consuming at all |  | Somewhat time-consuming |  | Very time-consuming |  | Don't know |  | No answer |
| --- | --- | --- | --- | --- | --- | --- | --- | --- | --- |

1. How time-consuming was it for you to attend the treatment appointments at the injection center?

|  | Not time-consuming at all |  | Somewhat time-consuming |  | Very time-consuming |  | Don't know |  | No answer |
| --- | --- | --- | --- | --- | --- | --- | --- | --- | --- |

1. How time-consuming was the organisation of injection appointments for you?

|  | Not time-consuming at all |  | Somewhat time-consuming |  | Very time-consuming |  | Don't know |  | No answer |
| --- | --- | --- | --- | --- | --- | --- | --- | --- | --- |

1. Have you ever had to cancel or skip an appointment with your eye doctor?

☐No ☐Yes, once ☐Yes, several times ☐Don't know ☐no answer

1. If yes: which appointment did you cancel / skip?

|  | Injection appointment |  | Appointment for a check-up following the eye injection |  | Appointment for an examination of my eyes/my vision/OCT examination |  | Don't know |  | No answer |
| --- | --- | --- | --- | --- | --- | --- | --- | --- | --- |

1. If yes: please give reasons for missed or cancelled appointments:

Lack of support from relatives or carers

☐ Lack of transport facilities

Other illness/s

Simply forgot the appointment

Other reason

Don't know

Not specified

1. **Mobility**
2. How do you get to the injection center or the post-injection follow-up practice?

☐ Car

☐ Public transport

☐ Taxi

☐ Other means of transport

☐ Don't know

☐ No answer

1. Did you need assistance from relatives or companions to attend your injection appointments? ☐Yes ☐No ☐Don't know ☐ No answer
2. Did you need support from relatives or friends to attend your check-up appointments?

☐Yes ☐No ☐Don't know ☐No answer

1. If yes: who supported you?

☐ Spouse

☐ Son or daughter

☐ Grandchild

☐ Friends

Other

No answer

1. If yes: did you need assistance with travelling to and from your destination?

☐Yes ☐No ☐Don't know ☐No answer

1. If yes: did you need someone to accompany you to the appointment?

☐Yes ☐No ☐Don't know ☐No answer

1. Would a taxi voucher provided by the health insurance company influence your decision to continue therapy?

|  | Completely |  | Rather yes |  | Rather no |  | Not at all |  | Don't know |  | No answer |
| --- | --- | --- | --- | --- | --- | --- | --- | --- | --- | --- | --- |

1. How long does it take you to get to the pot-injection follow-up center?

Less than 30 minutes

☐ 30-60 minutes

☐ More than 60 minutes

☐ Don't know

☐ No answer

1. How long does it take you to get to the control center?

Less than 30 minutes

☐ 30-60 minutes

☐ More than 60 minutes

☐ Don't know

☐ No answer

1. **Comorbidities**
2. Do you suffer from any other illnesses in addition to your eye(s) for which you are receiving long-term treatment? ☐Yes ☐No ☐Don't know ☐No answer
3. If yes: Which?

Diabetes

☐ Cardiovascular disease

☐ High blood pressure

☐ Joint disease (e.g. arthrosis, arthritis)

☐ Thyroid disease

☐ Mental illness (e.g. depression)

☐ Don't know

☐ Other

☐ N/A

1. How often do you see a doctor for other illnesses?

Several times a week

☐ 1x/week

☐ Every 2 weeks

☐ Every 4 weeks

☐ Not at all

☐ Don't know

☐ Not specified
